# Initial Standardized Framework for Reporting Social Media Analytics in Emergency Care Research

**DOI:** 10.5811/westjem.2018.3.36489

**Published:** 2018-05-15

**Authors:** Damian Roland, Jesse Spurr, Daniel Cabrera

**Affiliations:** * SAPPHIRE Group, Health Sciences, Leicester University, Leicester, United Kingdom; † Paediatric Emergency Medicine Leicester Academic (PEMLA) Group, Children’s Emergency Department, Leicester, United Kingdom; ‡ Redcliffe Hospital, Nursing Education and Emergency Department, Brisbane, Australia; § Metro North Hospital and Health Service, Brisbane, Australia; ¶ Mayo Clinic, Department of Emergency Medicine, Rochester, Minnesota

## Abstract

The use of social media platforms to disseminate information, translate knowledge, change clinical care and create communities of practice is becoming increasingly common in emergency and critical care. With this adoption come new lines and methods of inquiry for research in healthcare. While tools exist to standardize the reporting of clinical studies and systematic reviews, there is no agreed framework for examining social media–based research. This article presents a publication and appraisal checklist for such work and invites further collaboration in the form of a Delphi technique to clarify, expand, improve, and validate the proposal.

## INTRODUCTION

Utilization of social media, defined by the *Oxford English Dictionary* as “websites and applications that enable users to create and share content or to participate in social networking,” can potentially produce enormous data sets of information exchange between users and communities on online platforms. Social media clearly has an impact on healthcare,^1^ and healthcare research performed and analyzed through social media metrics has become increasingly recognized.^2^–^4^ Data may be extracted from social media platforms to demonstrate knowledge translation, education, or patient engagement; evaluate communication; and undertake real-time disease surveillance.^5^,^6^

As familiarity grows, social media is becoming valued as a source for insight into complex distributed systems, such as healthcare networks and communities of practice.^7^ In particular, the emergency and critical care communities have embraced social media as a means to reduce the knowledge translation gap.^8^–^10^ In their seminal review of the different mechanisms available to perform research on social media, Edwards et al.^11^ described the complexity of social research and highlighted the promise of social media to allow a previously impractical task: examining the real-time communications and interactions of a community through pre-defined methods of analysis, in volume large enough to draw generalizations. Edwards^11^ explained this by overlaying the features of intensive or extensive, and real-time or time-bound domains (Figure). “Intensive” methods such as ethnography (real-time) and interviews (time-bound) offer deep insights into communities but have limited generalizability. “Extensive” methods such as surveys and experiments offer potentially greater generalizability at the cost of being time bound.

The promise of large-scale, social media research lies in the domain of extensive and real-time features, and this has driven the development of new analytics platforms and softwares (termed “engines” for the proposed framework).^11^ We believe this constitutes one of the cornerstones for understanding the use of social media metrics in research. The use of “engines” for research presents a major gap in current guidance for reporting or critique of social media research.

Previously, research obtained through social media channels has been met with some skepticism from traditional scientific bodies. In part, this is due to lack of familiarity with the nature of the data, paucity of standard data extraction and analytical tools, and heterogeneous reporting systems.^12^ The ability to use consistent and reliable data abstractions, as well as standardize the data output, is important to the assessment for validity and applicability of social media research.^13^

Social media analysis is commonly understood as the amount of times a particular object, such as a member of a network or its interactions (e.g., an individual tweet or Facebook post or Instragram user) has been accessed, for example, “liked” or shared by others users. These metrics are often reported in the media. However, analysis is now far more complex and can look at aggregated objects over time, the interactions between users, and how communities develop within social media platforms.^7^ To demonstrate the impact of social media, particularly on patient outcomes, understanding the methodology will be vital for objective appraisal of novel work. This article represents a call to action to develop standard methodology for the use of social media analytics in emergency care research, and an interim framework for critical appraisal of published research in this arena.

## METHODOLOGY

To develop an initial framework for the use and reporting of social media analytics in emergency care research, we looked to established reporting guidelines for previous research. These mirror the Preferred Reporting Items for Systematic Reviews and Meta-Analyses (PRISMA)^14^ and others collated by EQUATOR (Enhancing the QUAlity and Transparency Of health Research).^15^ We propose this framework, recognizing the challenge of a standard approach to examining social media, using modern analytics to describe digital communities of practice in healthcare.^7^ Also, we note that none of the 319 reporting guidelines listed by the EQUATOR Network^15^ are concerned with research in social media.

The complexity and volume of data obtained from social media platforms have led to the development of numerous reporting tools, referred to as “engines” in our proposed framework, which simultaneously collect, curate, cross-reference and analyze data, presenting the end user with a refined and cleaned, or filtered, version. The analysis of social media data adds a significant layer of complexity because many of the mining and analytical tools are based at least in part on proprietary formulas and software. In light of this complexity and capacity for selective sampling, time-bound or retrospective sampling creates problems in rigor and reproducibility. We currently lack a methodology to examine whether research reporting on electronic data from a social media platform is robust, reliable, and valid. While the construct and design of social media research is different from basic^16^ and clinical science, there is no reason why social media research should not at least have best practice guidance – something which can be iteratively developed and applied for critical appraisal.

## INTIAL FRAMEWORK RECOMMENDATIONS

We framed our recommendations using the template developed by the PRISMA^14^ group for the reporting of systematic review data, as both data sets and analysis represent variations of knowledge synthesis.^16^ In social media research, the general aim is to describe a set of data related to an object (e.g., hashtag, social media user) in either a quantitative manner related to actions, such as sharing or accessing, or in a qualitative review of narrative content of the object (for example, sentiment or language analysis of the words used to describe a concept). The objective of this form of research is to uncover the knowledge held within a particular network and display that in an analytical fashion. This process needs to be well defined (similar to other forms of research); otherwise it is possible to purposefully select data in a biased manner and in large quantity to support or refute any thesis.

The PRISMA systematic review tool was chosen as a conceptual template, as the data sources for reviews can be heterogeneous, very similar to the data obtained from social media. Furthermore, the domains determining data quality in PRISMA mapped closely to those needed for extraction and analysis from social media sources.^7^,^16^ Our broad expectation is that the following format would be used to frame any scientific work around social media analysis: Title and Abstract, Introduction, Methods, Results, Conclusions, Disclosures.

Within Introduction we have developed a framework, resembling the Population, Intervention, Control, Outcome (PICO) format, for defining the objectives of the study, which includes a description of the Network (the social media platform being studied, e.g., Twitter); Object (the item of the network being studied, e.g., hashtag such as #FOAMed); Engine (proprietary data extraction and/or analytics platform), Comparison (secondary object or outcome for comparison), Observation (the theoretical lens and methodology for analysis, e.g., discourse analysis of Twitter conversations or descriptive quantitative measures such as volume and users). We propose the use of the acronym NOECO = Network, Object, Engine, Comparison, Observation. The recommendations for using this format are described in Table 1.

In accompaniment, Table 2 demonstrates the application of the framework to an example social media publication from the field of plastic surgery.^17^ In this paper the data analysis (Section no.11), synthesis of results (no.12) and data corruption (no.14) were not clearly defined or explained. This means that spam bots (computer-generated personas using soft artificial intelligence) may have been included in the analysis of data; the mechanism of evaluation by the social media engine is not clear; and the statistical analytical tool was not defined. These obviously may impact on the validity of the results and make it difficult to reproduce the evaluation undertaken.

## DISCUSSION

The original PRISMA guidance reflects the consensus of experts in the field of evidence-based practice. Currently there is no clearly defined “evidence” base for the interpretation of social media analytics that relate to healthcare interventions, improvements, or observations. As more literature is published in this growing field, it is important that the same standards be applied to evaluation of arguments or hypotheses in social media-based studies as in clinical trials. Our initial framework, particularly the NOECO objectives, promotes debate in this area.

As with the original PRISMA document,^18^ it is likely that evolution in social media analytics will require persistent and regular updates and derivatives to keep pace with advances in the field. We suggest that our checklist be publicly available and editable in the same way as Wikipedia^19^ to allow ongoing innovation in its design and application. The desired extension of this work is to seek collaboration from emergency care researchers and beyond to develop a best-practice consensus framework, likely through the use of a Delphi methodology.^20^

## Figures and Tables

**Figure:**
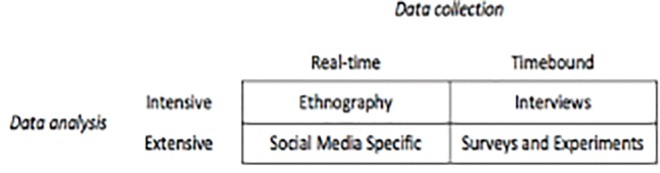
A matrix of data collection and analysis. Adapted from Edwards et al.^11^

**Table 1:** Checklist for publication of social media–based research, the NOECO statement.

Section/topic	No	Item	Page reported
Title	1	Identify manuscript as an analysis of social media data using specific analytical tools.	
Abstract			
Summary	2	Report the background; objectives: including the data source and time frame; methods: including analytical engine to extract the data as well as data management tools; results: description of raw data, description of post-analysis data and limitations; conclusions: key findings.	
Introduction			
Rationale	3	Describe what is already known about the topic and the rationale for the data extraction and analysis.	
Objectives	4	Provide an explicit statement of questions being addressed with reference to defining the network and what is being evaluated, compared, and observed (**NOECO – Network, Object, Engine, Comparison, Observation**). **Network**: Defined as the digital platform where users (nodes) share resources such as data. Examples include Facebook and Twitter. **Object**: Defined as the component of the network to be studied. It can consist of the users of the network, a particular set of data shared among them of the structures created by these interactions. Examples include tweets around a hashtag, sharing of a particular content or sentiment analysis on a particular population. **Engine**: Defined as a networks analysis tool used to measure the objects. This is typically a proprietary software able to mine and analyze large amounts of data. Examples include NodeXL and Gephi. **Comparison**: Defined as the comparison against which the measure is made, similar to comparison between intervention and control groups. Examples include the number of interactions between users in Twitter compared to the same users in Facebook. **Observation**: Defined as the actual observation hypothesized for the study. Examples include healthcare users of Twitter that are clustered around few sources.	
Methods			
Protocol	5	Indicate whether a protocol (i.e., a pre-defined method to undertake the evaluation of the social media data) exists, if it was created prior to the data extraction and analysis, and where it can be accessed (e.g. permalink at website).	
Data source	6	Describe the data source in terms of platform and type of data (e.g., raw data, filtered by the researchers, or managed by platform automatically).	
Data appropriateness	7	Describe theoretical frameworks, characteristics of the data, inferences about data, and inferences about users. (e.g., does the data that is suggested to be used have internal validity for the question that is being asked.)	
Data inclusion	8	Describe data to be included and search strategy to be used and rationale.	
Data exclusion	9	Describe data to be excluded, nodes or uses to be excluded, (e.g., suspected spam [automatic commercial offerings] or bots [automatic nodes designed to influence networks]), and data arguments to be excluded and rationale.	
Data extraction	10	Describe data extraction engine to be used, program interface version if available, output format, and corruption data percentage. Describe how data was filtered.	
Data analysis	11	Describe analytical tool used, cite pertinent papers describing methods of the tool, and describe the output format of the data. If analysis is performed by the data extraction engine itself, the underpinning (e.g., network centrality calculation – who/what are the most important people or nodes in a network) methodology should be described.	
Synthesis of results	12	Describe the statistical analysis tool (e.g., univariate analysis), specifically if using large datasets statistical tools (e.g., eigenvectors).	
Results			
Data selection	13	Provide platform, dates, and magnitude of the data points and search strategy.	
Data corruption	14	Provide magnitude of data corruption, contamination (spam bots), unobtainable or missing data. Describe source of corruption/bias.	
Data quality	15	Describe whether the data quality is appropriate in terms of size, corruption and ability to make appropriate inferences. Describe whether the Objects and Engine (from NOECO) were appropriate.	
Analysis	16	Describe how the data analysis supports or disproves the original question. Describe whether end points or surrogate markers were met. Describe the Comparison and Outcomes from the NOECO question.	
Discussion			
Summary	17	Describe the main findings in the dataset, i.e., how they do (or do not) answer the NOECO data question.	
Limitations	18	Describe data source, set, and analysis limitations.	
Conclusions	19	Provide a general interpretation of the data question after the data analysis.	
Disclosures	20	Describe sources of funding, support, and conflict of interest, particularly regarding proprietary data extraction and analysis tools.	

**Table 2:** Example of best practices for reporting and analysis on Branford OA, Kamali P, Rohrich RJ, et al. #PlasticSurgery. *Plast Reconstr Surg.* 2016;138(6):1354–65. Checklist items defined in Table 2.

Checklist item	Description on the paper	Page
1	The manuscript identifies itself implicitly as an analysis of social media data using the hashtag symbol in the title; however, it fails to specify analytical tools	1/1354
2	The manuscript reports background, objectives, data source (Twitter), description of the raw data, description of post analysis and conclusions. The abstract does not describe time frame, analytical engine, management tools or limitations.	1/1354
3	The article describes a round rationale of what is already known, particularly for the field of social media and plastic surgery.	1,2/1354–1355
4	The manuscript describes the objectives using a clear framework: Network: Twitter Object: Hashtag “Plastic-Surgery” (#Plastic-Surgery) and free text “plastic surgery” Analytical Engine: Not explicitly described, but appears to be Symplur Signals per citation in the references section. Comparison/Control: None apparent; this appears to be a descriptive netnographic analysis. Observation: Clearly described: hashtag-use description, subject matter, links to plastic surgery journals and self-promotion.	2–3/1355–1356
5	No description of protocol for data extraction.	
6	The manuscript describes network source, type of data and filters.	2/1355
7	The manuscript describes characteristics of the data, surrogate markers, inferences about producers and users.	2/1355–1357
8	Description of data inclusion is clear.	2/1355
9	Description of data exclusion is clear (e.g., bots and non-English).	2/1355
10	Not described, but inferred from references and figures to be Symplur Signals. No details on data corruption or refinement method.	
11	Not described.	
12	Not described.	
13	The manuscript describes platform, dates and data points clearly.	3/1356
14	Not described.	
15	NOECO statement described previously, and there is an implicit assertion that it was appropriate for the analysis.	
16	The manuscript contains a clear analysis about the data supporting the original study aim (description of the hashtag use).	3–11/1356–1364
17	The manuscript describes the main findings that answer the NOECO question.	3–11/1356–1364
18	No clear description on limitations.	
19	The manuscript provides a general interpretation of the data source, set and analysis.	11–12/1364–1365
20	The manuscript describes clear disclosures, including support and conflicts of interest.	1/1354
